# Antimicrobial-resistant organism carriage and metagenomic characterization of the microbiome and resistome in veterinary workers

**DOI:** 10.1007/s11259-026-11516-6

**Published:** 2026-09-16

**Authors:** Laurel E. Redding, Weiming Hu, Scott Daniel, Maho Okumura, Maeve E. Hiehle, Michael Z. David, Lynn Hunter, Stephen D. Cole

**Affiliations:** 1 Department of Clinical Studies – New Bolton Center, School of Veterinary Medicine, University of Pennsylvania, Kennett Square, PA USA; 2 Division of Gastroenterology, Hepatology, and Nutrition, Children’s Hospital of Philadelphia, Philadelphia, PA USA; 3 Department of Pathobiology, School of Veterinary Medicine, University of Pennsylvania, Philadelphia, PA USA; 4 Division of Infectious Diseases, Department of Medicine, University of Pennsylvania, Philadelphia, PA USA

**Keywords:** Antimicrobial resistance, Staphylococcus aureus, Escherichia coli, Microbiome, Resistome, Veterinary

## Abstract

**Background:**

Veterinary personnel work in environments with high exposure to antimicrobials and antimicrobial-resistant pathogens. However, the extent to which these occupational exposures influence their carriage of antimicrobial-resistant (AMR) organisms and the composition of their microbiome and resistome remains poorly understood. The objective of this study was to characterize AMR pathogen carriage and microbiome/resistome composition in veterinary personnel and to assess whether workplace setting and patient-related exposures were associated with detectable differences in these outcomes.

**Methods:**

Personnel from a large academic veterinary hospital and a convenience sample of private-practice small-animal veterinary hospitals provided nasal swabs, stool samples, and exposure survey data; environmental samples were also collected from the academic hospital. Nasal swabs were cultured for methicillin-resistant *Staphylococcus aureus*, and stool samples were cultured for extended spectrum cephalosporin resistance (ESCR) *Escherichia coli*. Samples also underwent shotgun metagenomic sequencing or 16S rRNA gene sequencing for taxonomic and antimicrobial resistance gene profiling. Alpha/beta diversity were compared across groups, beta diversity by Permutational Multivariate Analysis of Variance, linear models tested differential abundance, and logistic regression was used to assess covariates associated with MRSA and ESCR carriage. A subset of academic-hospital participants also submitted additional samples after time away (1–2 weeks) from the hospital.

**Results:**

Among academic-hospital personnel (n = 38), MRSA and ESCR-*E. coli* carriage prevalence were 18.4% and 11.1%, respectively. In private-practice personnel (n = 8), carriage was 12.5% and 25%, respectively. Within the academic hospital, MRSA carriage was associated with administering antibiotics to patients. Microbial composition differed modestly by worksite (R^2^ = 0.02) when assessed using unweighted UniFrac metrics only, suggesting subtle site-related differences in low-abundance phylogenetically distinct taxa. Global resistome composition did not differ significantly by site or clinical exposure, although a small number of individual AMR ontologies and broad AMR gene families varied across groups, including those associated with commonly-administered oral antibiotics at the hospital. Paired on/off-clinic analyses did not show a clear directional shift after time away from the hospital.

**Conclusions:**

Veterinary personnel carried MRSA at a notable frequency, while workplace effects on the microbiome and resistome were subtle, with limited evidence for large-scale shifts in community structure by site or patient exposure.

**Supplementary Information:**

The online version contains supplementary material available at https://doi.org/10.1007/s11259-026-11516-6.

## Introduction

Antimicrobial resistance (AMR) is a persistent and evolving challenge in veterinary hospitals. Animals are routinely treated with antimicrobials, and infected animals can contaminate the hospital environment, where resistant organisms may persist on surfaces and fomites (Sebola et al. 2023). Veterinary personnel working in these hospitals are frequently exposed to colonized or infected animals, contaminated surfaces, and high-risk procedures involving feces, blood, and other bodily fluids.

Despite these exposures, relatively few studies have characterized antimicrobial-resistant organism carriage among veterinary healthcare workers. Methicillin-resistant *staphylococci* (MRS) and extended spectrum cephalosporin-resistant *Enterobacterales* (ESCRE) have been detected in veterinary personnel (Hanselman et al. 2006; Espadale et al. 2018; Meijs et al. 2021; Dierikx et al. 2022), and a small number of studies suggest that veterinary workers may carry these antimicrobial-resistant organisms at a higher prevalence than the general population (Royden et al. 2019; Meijs et al. 2021; Dierikx et al. 2022). However, most available data focus on individual pathogens and do not assess the broader gastrointestinal resistome (collection of AMR genes) or microbiome composition of veterinary personnel across practice types and levels of clinical exposure. Understanding colonization patterns and factors that influence the composition of the microbiome and resistome in veterinary workers is important for better understanding veterinary occupational health. Notably, because colonization is associated with increased risk of subsequent infection (Davis et al. 2004; Platteel et al. 2015; Nelson et al. 2019), it is important to understand whether and how often veterinary workers carry AMR bacteria. While veterinary personnel represent an actively employed workforce and thus may differ from the general population in ways consistent with the healthy worker effect (Li and Sung 1999), they may be vulnerable to infection when occupational exposures result in bites, kicks, scratches, or other breaches in skin integrity. Additionally, in a predominantly female population of child-bearing age, individuals may experience periods of immunosuppression during pregnancy that could predispose to infections. Moreover, because colonized individuals or individuals with expansive resistomes could act as a node for dissemination of AMR organisms within hospitals (including, possibly, transmission to patients), this information could have implications for infection control.

The objectives of this study were to characterize AMR organism carriage and gastrointestinal and nasal microbiome/resistome composition among veterinary personnel across multiple clinical settings and to assess the contribution of workplace exposures to these microbiological characteristics. We considered three hypotheses, including that 1) AMR organisms would be found among vet hospital workers at levels consistent with or higher than those found in the general population; 2) personnel with greater exposure to animal patients (and in particular, exposure to patients being treated with antibiotics) would be more likely to carry AMR bacteria and would harbor a more expansive resistome; and that 3) participants’ microbiome and resistome would cluster by worksite, consistent with acquisition of microbiota from a shared environment.

## Methods

### Participant population

Six veterinary hospitals were selected for inclusion in the study: a large animal academic veterinary hospital (“NBC”) and five small animal primary care or referral hospitals based on the authors’ personal network. The large animal academic hospital sees mostly horses and ruminants, and the most frequently used classes of antibiotics in this hospital were penicillins, cephalosporins, and tetracyclines (Redding et al. 2019). The small animal hospitals see almost exclusively dogs and cats, but no data were available on their routine antibiotic use.

All hospital personnel at our academic hospital for large animals were invited to participate in the **ST**udying **R**esistance **I**n **V**eterinary **E**nvironments (STRIVE) study by email. Primary contacts of the investigators at the small animal hospitals were contacted by the investigators and asked to disseminate the invitation to participate in the study among their hospital staff. Participants agreeing to participate self-collected a stool sample in a sterile collection container and two nasal swabs (ClassiqSwabs, Copan Diagnostics, Murrieta, CA) and completed a survey querying participant-level information, including demographics, role in the hospital, degree and types of contact with patients on the prior day and prior week, and locations visited in the hospital during the same time period. Participants were instructed to collect their samples on the same day (February 13, 2025), and samples were collected from a central drop-off location in the hospital on February 14, 2025 and immediately stored at -80 °C. One swab was used for targeted organism detection, the other for sequencing.

During the same time period, six pooled swabs of the NBC hospital environment were taken from different areas of the academic hospital, including 1) surgical patient barns, 2) general patient barns (horses and small ruminants), 3) the hospital clinical area, 4) the food animal barn, 5) the colic hospital wing, and the 6) isolation hospital wing. Surfaces sampled included aisles, stall doors, stall drains, hand surfaces, and floors. All swabs underwent culture for targeted pathogen detection but only four environmental samples contributed to metagenomic analyses due to loss of one sample and insufficient reads following sequencing in another sample.

To assess whether colonization status and microbiome/resistome composition changed with time away from the hospital, participants were invited to submit a second set of samples if they planned to be away from the hospital (e.g., for travel, time off, vacation, etc.) for at least a week in the months following the initial sampling date. The subset of participants who agreed to participate were provided with the same set of sample collection supplies and instructed to collect their samples the day before returning to work.

To assess the effect of site and practice type on colonization of personnel, a convenience sample of veterinarians and veterinary technicians from private practices in the greater Philadelphia area were also recruited through the authors’ personal network. Supplies for sample collection (nasal swab and stool sample) were sent to personnel agreeing to participate along with shipping containers and pre-paid shipping labels for overnight shipping. Participants were instructed to complete an adapted version of the survey on the same day they collected their sample. Once samples were received, they were first processed for cultures and subsequently stored at -80 °C for molecular analyses. This study was approved by the Institutional Review Board of the University of Pennsylvania (Protocol 857,637).

### Staphylococcus aureus culture

Nasal swabs were processed using established protocols. Briefly, swabs were plated directly on *S. aureus*-selective solid media (Chromagar *S. aureus*, CHROMagar, Paris) and incubated at 37 °C for 24–48 h (“A” culture), while the swab tip was concurrently incubated in 5 mL tryptic soy broth (TSB) at 37 °C overnight (“B” culture). If *S. aureus* growth was observed on the “A” culture, that isolate was used; if not, 10 µL of the “B” culture was plated on SA Chromagar to recover any viable *S. aureus*. *S. aureus* colonies obtained from either agar or broth cultures were isolated, frozen at − 80 °C (single-colony and pooled stocks), and subsequently plated on Chromagar MRSA (CHROMagar, Paris), with observed growth determining methicillin resistance. Confirmation of the identity of isolates with other biochemical or molecular tests was not pursued, but our experience with human colonization cultures using Chromagar *S. aureus* followed by whole genome sequencing has shown only 3–4 of > 4000 positive cultures has resulted in isolation of a bacterium other than *S. aureus* (unpublished data).

### Escherichia coli culture and ESCR-E. coli selection

Fecal specimens were mixed using a sterile cotton tipped applicator and approximately 1–2 g removed for culture. Specimens were enriched in 5 mL of MacConkey broth (Northeast Laboratory Services, Winslow) and incubated for 16–24 h at 35 °C. A 10 ul and 100ul subculture were performed to MacConkey and HardyChrom ESBL agar (Hardy Diagnostics, Santa Maria, CA) respectively and incubated under the same conditions. Growth was evaluated the next day and all unique morphologies from the MacConkey were subcultured and speciated by MALDI-TOF analysis (Sirius Biotyper, Bruker, Billerika). Bacteria consistent with *E. coli* on the chromogenic ESBL agar (pink to magenta colonies) were subcultured and identity confirmed via MALDI-TOF. All isolates identified as *E. coli* underwent antimicrobial susceptibility testing using the Vitek 2 (Biomerieux, Paris) and the GN66 cards according to manufacturer instructions. The VITEK 2 GN66 card included the following antimicrobial agents: amoxicillin-clavulanic acid, ampicillin, ampicillin-sulbactam, piperacillin-tazobactam, cefazolin, cefoxitin, cefepime, ceftazidime, ceftriaxone, aztreonam, ertapenem, imipenem, amikacin, gentamicin, tobramycin, ciprofloxacin, levofloxacin, nitrofurantoin, and trimethoprim-sulfamethoxazole. The card also included an ESBL confirmation test. Manufacturer recommended quality control was performed per lot of cards. Interpretations were based on current CLSI standards and ESCR was defined as resistant to either ceftazidime and/or ceftriaxone (CLSI 2026).

### Shotgun metagenomics

#### DNA Purification, library preparation and sequencing

Stool samples (n = 52), nasal swabs (n = 37), and hospital environmental samples (n = 5) underwent shotgun metagenomic sequencing to characterize taxonomy and antimicrobial resistance within the study populations. DNA was extracted from 200 mg of stool using the Qiagen Power Soil DNA Extraction Kit (Qiagen, Hilden, Germany) then quantified using the Quant-iTTM PicoGreen dsDNA assay kit (Thermo Fisher Scientific) before library generation. For fecal and environmental samples, shotgun libraries were generated from 7.5 ng DNA using Illumina DNA Prep Library Prep kit and IDT for Illumina unique dual indexes at 1:4 scale reaction volume. Library success was assessed by Quant-iT PicoGreen dsDNA assay and samples with library yields < 1 ng/μl were re-prepped as needed. After all samples for a given pool were prepped, an equal volume of library was pooled from every sample and then the pool was sequenced using a 300 cycle Nano kit on the Illumina MiSeq. Libraries were then repooled based on the demultiplexing statistics of the MiSeq Nano run. Final libraries were QCed on the Agilent BioAnalyzer to check the size distribution and absence of additional adaptor fragments. Libraries were sequenced on an Illumina Novaseq 6000 v1.5 flow cell (Illumina, San Diego), producing 2 × 150 bp paired-end reads. Extraction blanks and nucleic acid-free water were processed along with experimental samples to empirically assess environmental and reagent contamination. A laboratory-generated mock community consisting of DNA from *Vibrio campbellii* and Lambda phage was included as a positive sequencing control.

Nasal swabs underwent 16S rRNA gene sequencing, as shotgun metagenomic sequencing of nasal samples is complicated by the high levels of human DNA in samples. Briefly, the V1-V3 region of the 16S rRNA gene was amplified using barcoded primers for use on the Illumina platform. Sequencing was performed using 250-base paired-end chemistry on an Illumina MiSeq platform.

Sequencing data were uploaded to NCBI under BioProject ID: PRJNA1474380.

### Bioinformatics processing

Metagenomic sequencing data was processed with the Sunbeam pipeline (v 4.7.0)(Clarke et al. 2019). Adapters were removed with Cutadapt (v4.9) (Martin 2011) and sequence quality filtering was performed using Trimmomatic (v 0.39) (Bolger et al. 2014) with recommended parameters. Reads with an overall complexity score less than 0.55 were removed using komplexity (v 0.3.5) (Clarke et al. 2019). Reads arising from human host or phi X 174 were removed by alignment to the human (GRch38) or phi X 174 genomes bwa-mem (v 0.7.18), using a minimum thresholds of 50% sequence identity and 60% aligned (Li and Durbin 2009). Taxonomic classification was then performed using Kraken2 (v 2.1.2)(Wood et al. 2019) with Unified Human Gastrointestinal Genome (UHGG, v 2.0.2)(Almeida et al. 2021) database standard version released on 2024–06-05 (https://www.ebi.ac.uk/metagenomics/genome-catalogues/human-gut-v2-0-2). To reduce false positive assignments in Kraken2, we set the ‘minimum hit group’ to 4 and ‘confidence’ to 0.05, as recommended by the software developers(Lu et al. 2022). Samples with less than 1 million classified reads were excluded for downstream analysis.

Reads were mapped to the CARD database (version 3.2.7) (Alcock et al. 2023) using Diamond (Buchfink et al. 2021) to estimate the abundance of antimicrobial resistance genes (ARGs). For ARG analyses, counts assigned to individual antibiotic resistance ontology terms (AROs) were normalized by the total number of reads mapped to CARD and by ARO length in kilobases. For downstream relative-abundance analyses, zero values were replaced by a small pseudocount corresponding to one-half of the minimum non-zero value observed for a given ARO, and abundances were then log-transformed for linear modeling. For some visualization and feature-selection steps, analyses were restricted to the most abundant genes or gene families present in at least 50% of samples. AMR gene family- and drug class-level abundances were generated by summing normalized abundances across AROs belonging to the same CARD-defined family or drug class.

For taxonomic analyses, relative abundances were calculated as the taxa count/(total count of microbial reads) of that sample, with only samples having more than 50,000 reads included in downstream analyses. For alpha richness calculations, the rarefaction was set at 100,000 reads after quality control, and Shannon diversity was calculated directly using microbial read counts.

For the 16S rRNA gene sequencing data from the nasal samples, sequences were demultiplexed using QIIME2 software (v. 19) (Caporaso et al. 2010), and denoised using DADA2 (Callahan et al. 2016). Sequences were aligned using Mafft (Katoh and Standley 2013) and phylogenetic reconstruction performed using Fasttree (Price et al. 2010). Mapping data with relevant sequencing parameters are included in Supplementary File 1.

### Analysis

For each organism, the prevalence of carriage among participants and locations was determined. Mixed effects logistic regression models were used to determine the association between colonization status and participant- and exposure-level factors, service, patient load, and procedures performed prior to sampling, considering hospital as a random effect.

For shotgun metagenomic data, between-sample community dissimilarity was assessed using Bray–Curtis and weighted and unweighted Unifrac distances and visualized by principal coordinates analysis (PCoA). Group-level differences in community composition were tested using PERMANOVA. Richness and Shannon diversity indices of all samples rarefied at 100,000 reads were calculated and compared by site, clinical exposure, and on/off clinic comparisons using linear models. Log10-transformed differential abundance of taxa, AROs, AMR gene families, and drug classes between groups was assessed using linear models; for paired on/off-clinic comparisons, linear mixed-effects models including participant as a random effect were used. P values were adjusted for multiple testing using the Benjamini–Hochberg false discovery rate procedure. R version 4.6.0, with packages including vegan, adonis2, nlme, emmeans, broom, mixed, taxafmt, tidyverse, ape, pheat, and ggplot2, was used to produce beta diversity, and linear tests and figures.

## Results

### Participants and carriage results

A total of 38 participants at our academic hospital participated, including 21 clinical veterinarians (13 faculty, eight house officers), 10 veterinary nurses (six OR/anesthesia, four barn), one barn crew member, and six non-clinical personnel (research or administration). Eight participants (six veterinarians and two veterinary technicians) from five private practice hospitals participated. Exposures to patients in the prior day/week among participants with clinical exposures varied in extent and frequency across participants, and all participants reported owning animals at home – mostly dogs and cats (Supplemental Files 2 and 3). The prevalence of *S. aureus* and MRSA was 36.8% (95% CI 21.8–54.0%) and 18.4% (95% CI 7.7–34.3%), respectively, among participants from the academic hospital, and 37.5% (95% CI: 8.5–72.5) and 12.5% (95% CI 3.2–52.7%), respectively, among private practice participants (Table 1). *E. coli* was isolated from the stool of all but two participants (94.7%) from the academic hospital, four (11.1%, 95% CI 3.1–26.0%) of which were ESCR-*E. coli*, six (16.7%, 95% CI 6.4–32.8%) of which were multi-drug resistant (resistant to at least three classes of antibiotics), and 18 (50%, 95% CI 32.9–67.1%) of which were resistant to at least one antibiotic. Among private practice participants, two (18.2%, 95% CI 3.1–65.1%) participants carried ESCR-*E. coli*, and seven (53.9%) and four (30.8%) carried *E. coli* isolates that were resistant to at least one or three or more antibiotics (i.e., multi-drug resistant), respectively.

**Table 1:** Number (%) of *E. coli* and *S. aureus* isolates obtained from different participant / sampling populations

	ESCR-*E. coli*	MDR *E. coli*	*Staphylococcus aureus*	Methicillin-resistant *S. aureus*
NBC participants	4/36 (11.1%)	6/36 (16.7%)	14/38 (36.8%)	7/38 (18.4%)
NBC participants – second round of samples	2/8 (25%)	4/8 (50%)	3/8 (37.5%)	1/8 (12.5%)
NBC environmental samples	2/6 (33.3%)	3/6 (50%)	1/6 (16.7%)	0/6 (0)
Private practice veterinary personnel	2/8 (25%)	4/8 (30.8%)	3/8 (38%)	1/8 (12.5%)

NBC = large-animal academic hospital; ESCR = extended-spectrum cephalosporin resistance; MDR = multi-drug resistant (resistant to 3 or more classes of antibiotics).

*E. coli* was isolated from all six of the sampled environmental sites, two (33.3%) of which demonstrated extended spectrum cephalosporin resistance (sites 2 and 4), and three (50%) of which were resistant to three or more drug classes. Only one site yielded *S. aureus* (methicillin-susceptible, Site 3). Carriage results as determined by culture were verified against shotgun metagenomics results for the AMR organisms: *S. aureus* was detected in four of the seven MRSA culture-positive samples, while the *mecA* gene was identified in all seven of the MRSA culture-positive samples. *E. coli* and the *OXA* gene were detected by shotgun metagenomics in all ESCR-*E. coli*-positive samples but no other beta-lactamases (*CTX, SHV, TEM*) were detected in any samples.

Among NBC participants, factors significantly associated with carrying *S. aureus* on multivariable analysis included owning a horse (OR = 20.9, p = 0.001, 95% CI = 3.52–124.5) and having worked the previous day in the food animal barn (OR = 12.6, p = 0.015, 95% CI = 1.65–96.0). Factors associated with MRSA colonization included having administered antibiotics to patients the previous day (OR = 14.4, p = 0.005, 95% CI = 2.2–94.0), having a greater number of animals/pets at home (OR = 1.2, p = 0.02, 95% CI = 1.0–1.3), and having animals at home that received antibiotics in the prior three months (OR = 17.3, p = 0.02, 95% CI = 1.6–190.7). The only variables significantly associated with carrying MDR *E. coli* was carriage of *S. aureus* (OR = 5.5, p = 0.023, 95% CI = 1.3–24.2) and visiting farms outside of work (OR = 6.3, p = 0.02, 95% CI = 1.3–29.3).

### Microbiome results

**Fig. 1:**
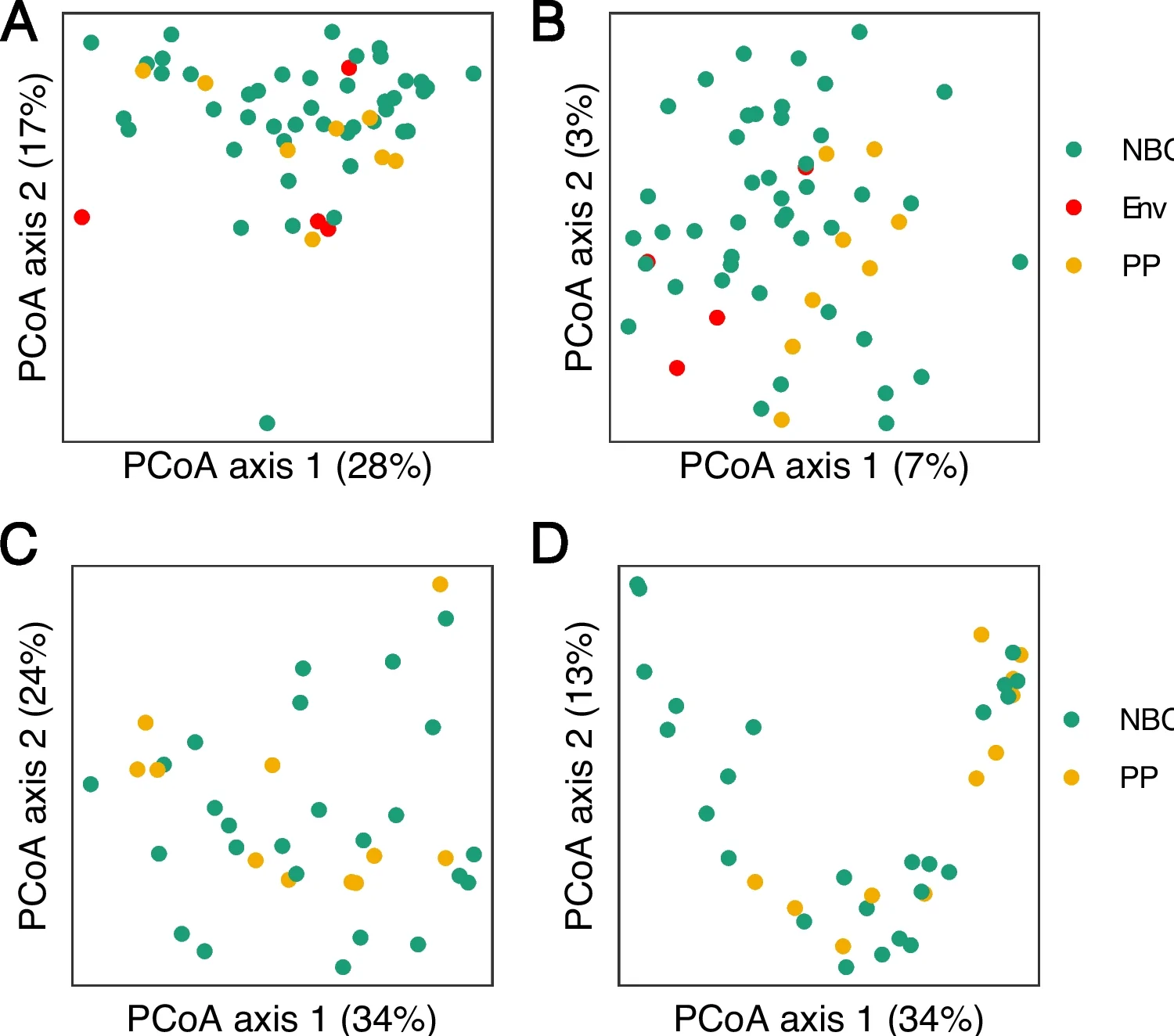
Principal coordinate analysis plots displaying UniFrac distances between samples from an academic large-animal veterinary hospital (NBC), its environment (Env) and from 5 small animal private practice clinics (PP). A: Weighted Unifrac distances of participants’ fecal microbiota. B: Unweighted Unifrac distances of participants’ fecal microbiota. C: Weighted Unifrac distances of participants’ nasal microbiota. D: Unweighted Unifrac distances of participants’ nasal microbiota

For both the fecal and nasal microbiota, we found that microbial composition differed significantly by work location when considering unweighted UniFrac beta diversity (PERMANOVA p < 0.05, R^2^ = 0.02), but not weighted UniFrac, Bray–Curtis, or Jaccard metrics for both fecal and nasal microbiome. No apparent clustering was visible on PCoA plots (Fig. 1). No other tested factors were statistically significantly associated with microbiota composition on PERMANOVA.

**Fig. 2:**
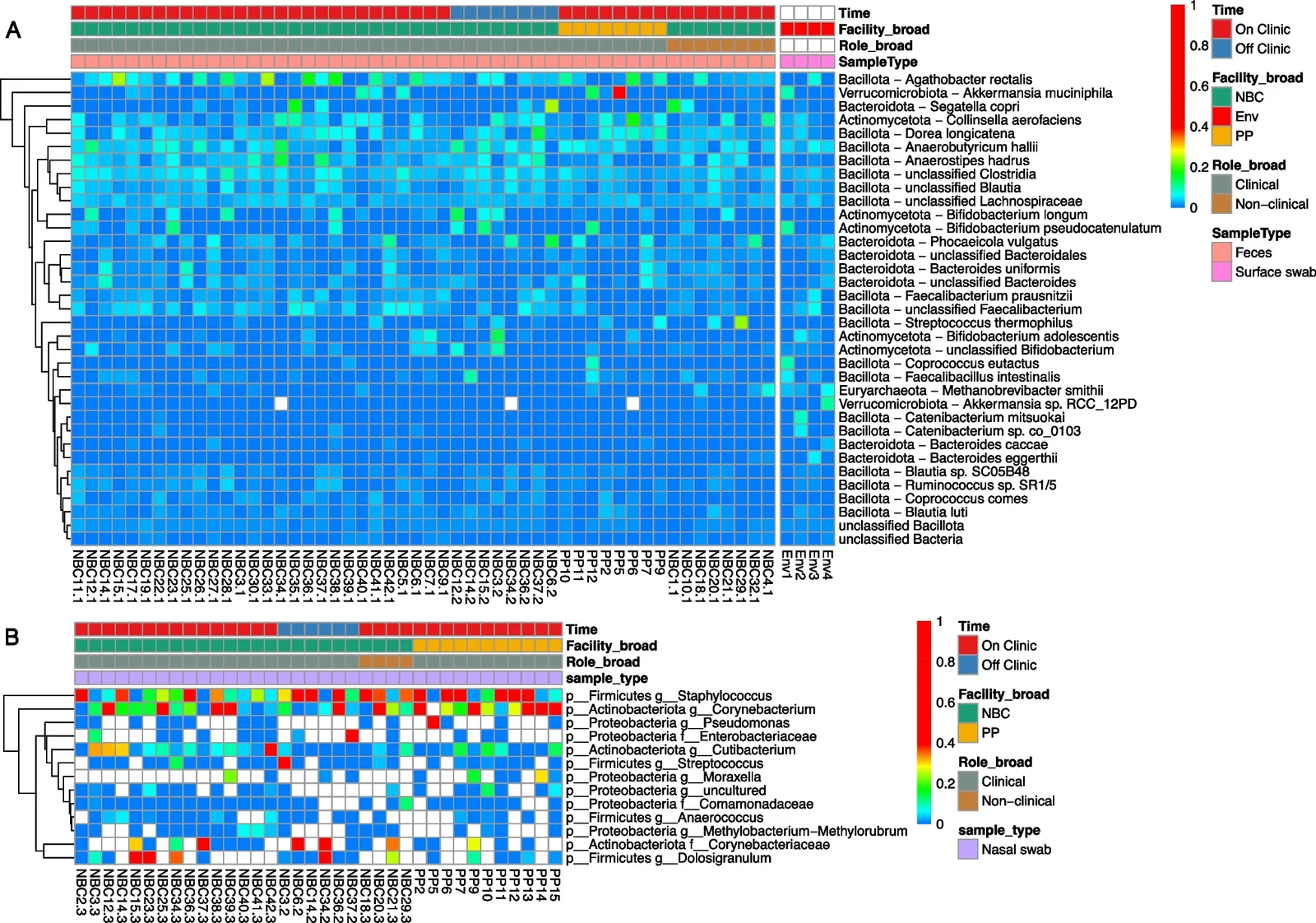
Heat plots displaying the relative abundance of taxa found at levels of at least 1% in samples from the fecal (**A**) and nasal (**B**) microbiota of veterinary personnel from a large-animal academic veterinary hospital (NBC) and 5 small-animal private practice clinics (PP)

No distinct site-specific taxonomic patterns were detectable, with the most commonly detected taxa encountered at varying levels in participants from all sites (Fig. 2). Overlap between environmental samples and fecal samples from NBC participants was noted, although a number of taxa were found at higher levels in the environmental samples, including *Catenibacterium* sp (mean relative abundance in environmental samples = 1.0%), *Akkermansia* sp (2.2%), and *Methanobrevibacter smithii (3.6%)*, and *Coprococcus eutactus (2.1%*).

There were three taxa that differed significantly in the fecal microbiota between work sites —*Faecalibacterium prausnitzii* (p < 0.0001, mean relative abundance = 1.3%), *Anaerostipes hadrus* (p = 0.01, mean relative abundance = 3.4%), and an unclassified *Faecalibacterium* (p = 0.02, mean relative abundance = 2.6%)*.* These taxa, well-established members of the healthy human gut and important butyrate producers, were generally found at higher levels among NBC participants. In the nasal microbiota, no taxa were found at significantly different levels comparing NBC and PP participants.

**Fig. 3:**
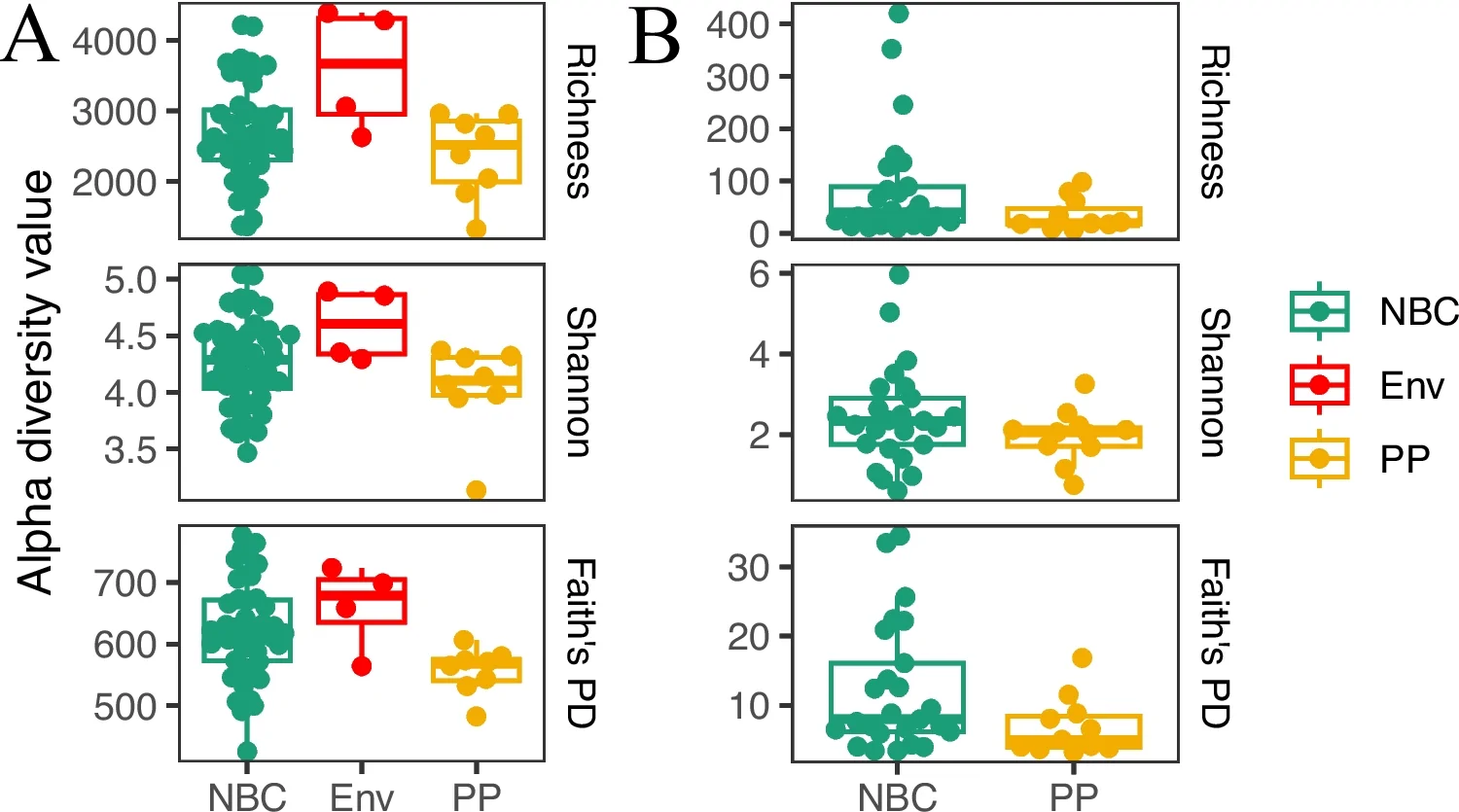
Alpha diversity of the gut (**A**) and nasal (**B**) microbiota of veterinary personnel from a large-animal academic hospital (NBC), its environment (Env), and five small animal private practices (PP)

A significant difference in Faith’s phylogenetic diversity between the microbiota of NBC and PP participants was observed for both the fecal and nasal microbiota, but no such difference was observed in richness or Shannon diversity (Fig. 3).

Among NBC participants, the only factor significantly associated with Shannon diversity of the fecal microbiota was recent exposure to patients with diarrhea and/or reflux: this exposure was associated with a 0.16-unit decrease in Shannon diversity (p = 0.014, 95% CI = -0.29 to -0.03). Factors associated with Faith’s diversity were having dogs at home (-82.0, p = 0.016, 95% CI = -148 to -15) and carrying ESBL *E. coli* (116, p = 0.001, 95% CI 49–181).

### Resistome results

**Fig. 4:**
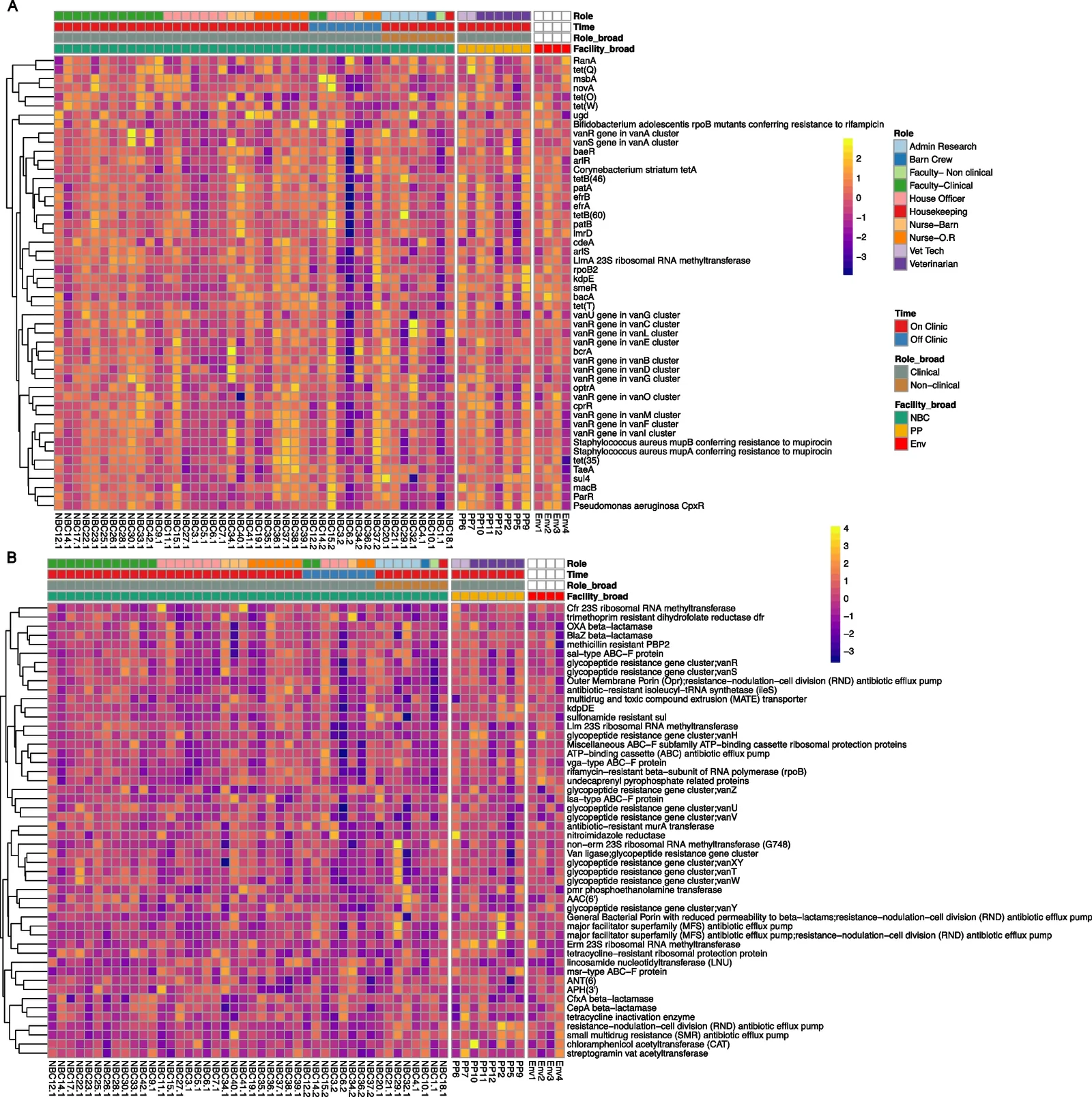
Heat maps displaying the distribution of the 50 most abundant AMR gene families (A) and antibiotic resistance gene ontologies (**B**) among veterinary personnel from a large-animal academic hospital (NBC), its environment (Env), and five small animal private practices (PP). Colors represent z-transformed log relative abundances

Across participants, dominant AMR gene families included efflux pumps (RND, MFS, ABC), porins, β-lactamase-related genes, glycopeptide (van) clusters, and ABC-F ribosomal protection proteins (Fig. 4A). Individual AROs (Fig. 4B) commonly included diverse tetracycline resistance genes, ABC-F (*lsa/vga/msr/optrA/poxtA*), efflux proteins (e.g., *macB, efrA/B, YojI*), and *vanR/vanS/vanH* cluster genes. No clear trends in the overall distribution of AROs or AMR gene families were noted by site or role within sites, and there was general overlap in the AROs encountered in environmental and participant samples. Four AROs were found to be at significantly lower levels in NBC participant samples compared to NBC environmental and PP samples, including *poxtA* (p = 0.006, mean RPKM = 10.9), *TxR* (p = 0.018, mean RPKM = 19.6), *YojI* (p = 0.019 mean RPKM = 11.9) and *CpxR* (p = 0.043, mean RPKM = 27.7). Four AROs were also found to be significantly higher in participants with clinical (patient) exposures compared to participants without patient contact, including *tet(T)* (p = 0.019, mean RPKM = 28.0), *dfrF* (p = 0.022, mean RPKM = 19.9), *vanS* (p = 0.037, mean RPKM = 10.5), and *vanH* (p = 0.046, mean RPKM = 10.3).

**Fig. 5:**
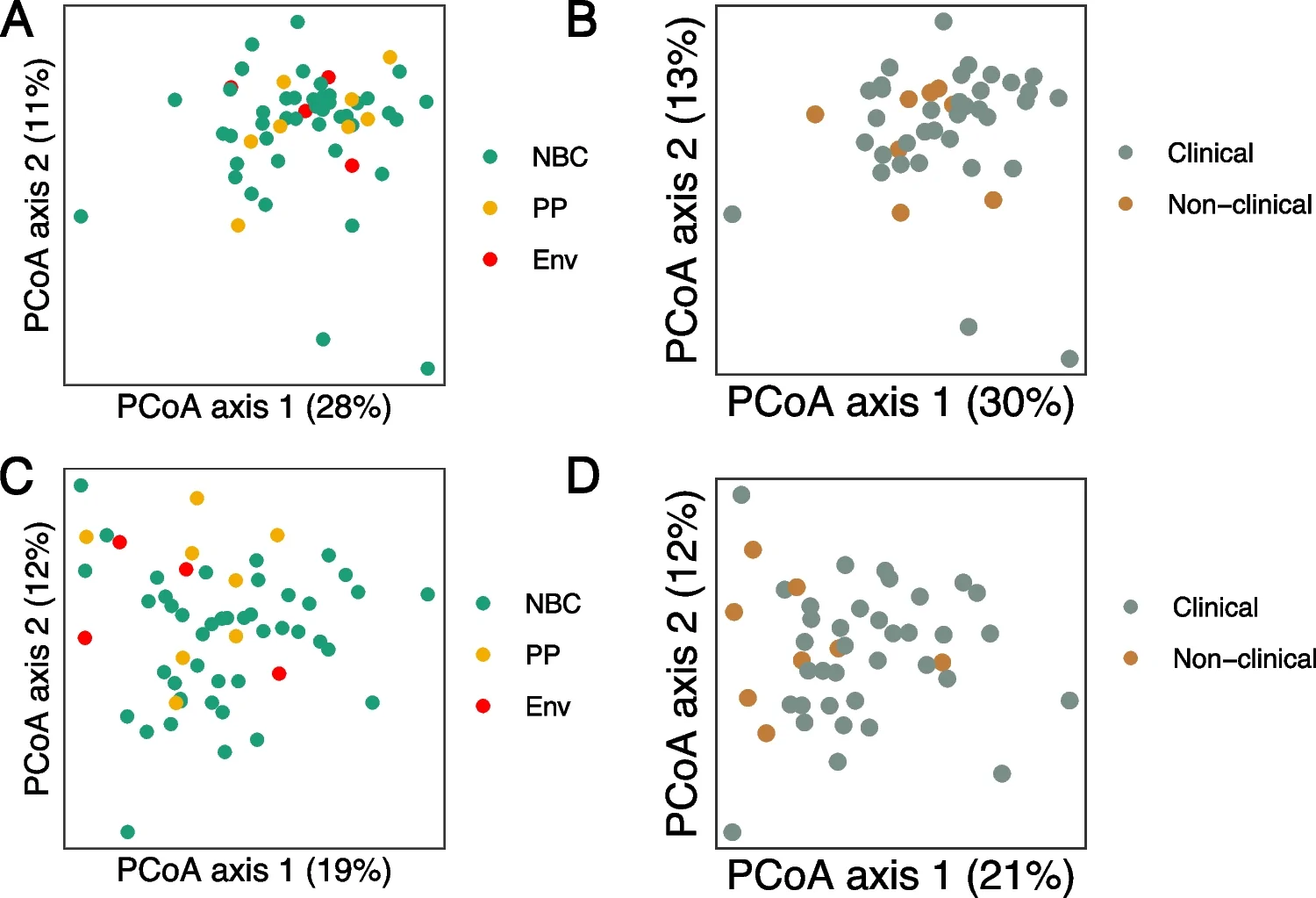
Compositional differences in the resistome by site (**A**, **C**) and clinical exposure (**B**, **D**) considering Bray–Curtis diversity of antibiotic resistance ontologies (**A** and **B**) and antimicrobial resistance gene families (**C** and **D**) among veterinary personnel from a large-animal academic hospital (NBC), its environment (Env), and five small animal private practices (PP). Clinical and non-clinical personnel are from NBC only.

When considering Bray–Curtis diversity metrics, no significant differentiation or clustering by site (Fig. 5A) or clinical exposure (Fig. 5B) was noted for the overall composition of the resistome (p = 0.48 on PERMANOVA) based on AROs.

**Fig. 6:**
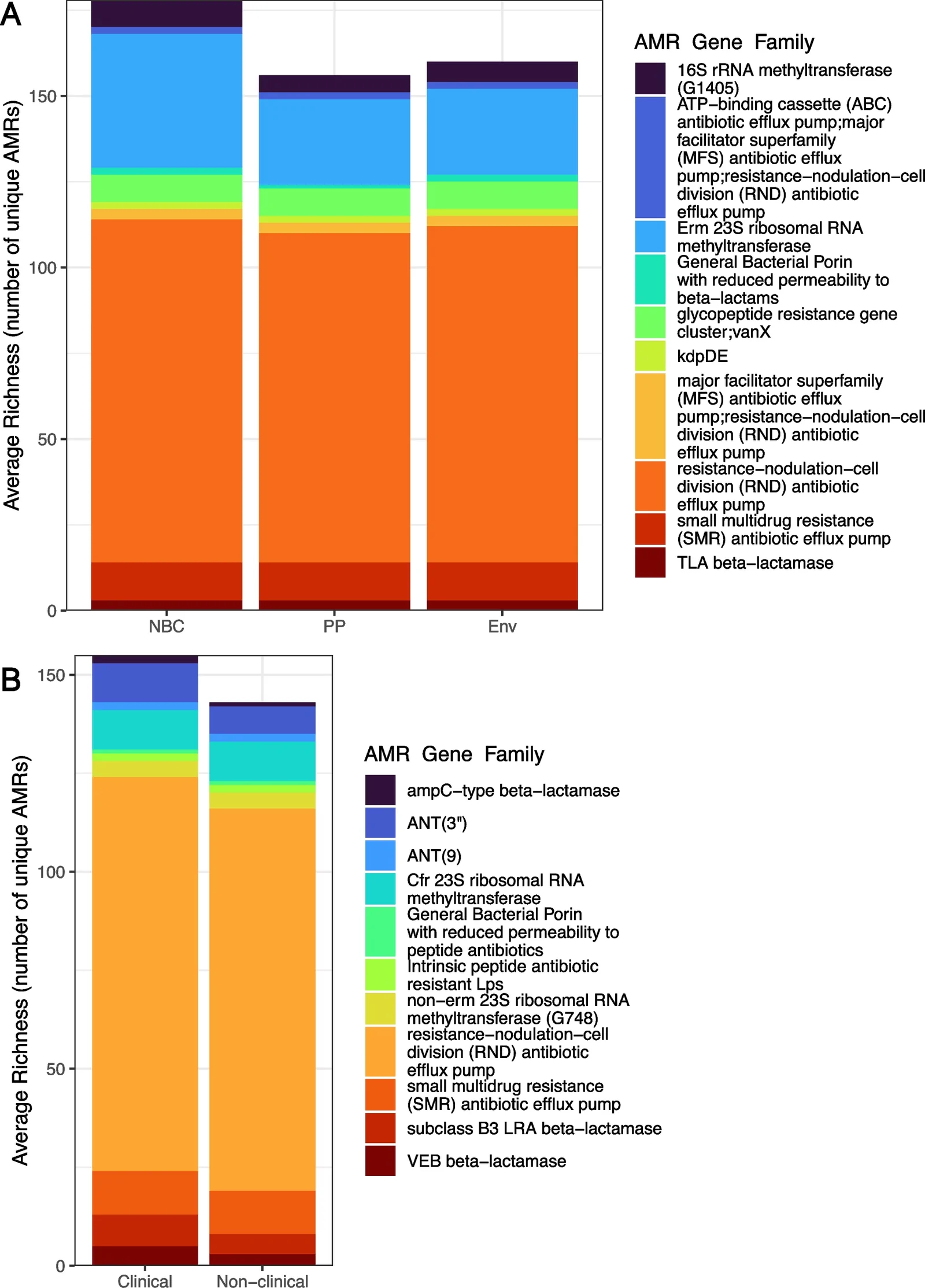
Average richness of antimicrobial resistance gene families by work site (**A**) and by exposure to patients (**B**) in veterinary personnel from a large-animal academic hospital (NBC), its environment (Env), and five small animal private practices (PP)

Several AMR gene families were found at different levels across sites (Fig. 6A) and clinical roles (Fig. 6B), but these differences did not reach statistical significance when adjusting for false discovery rates. These AMR families tended to belong mostly to the intrinsic, mechanistic, multi-drug families, e.g., ABC efflux, RND efflux, MFS + RND combinations, and porins.

### On vs off clinics

Among the 8 NBC participants who submitted a second sample following time away from the hospital (1–2 weeks), no stool samples carried ESCR-*E. coli* at any time point. One participant was *S. aureus*-negative at both time points, whereas three of the other participants who were initially *S. aureus*-negative became colonized with MSSA following time away from the hospital. One participant who was initially colonized with MRSA became colonized with MSSA following time away.

**Fig. 7:**
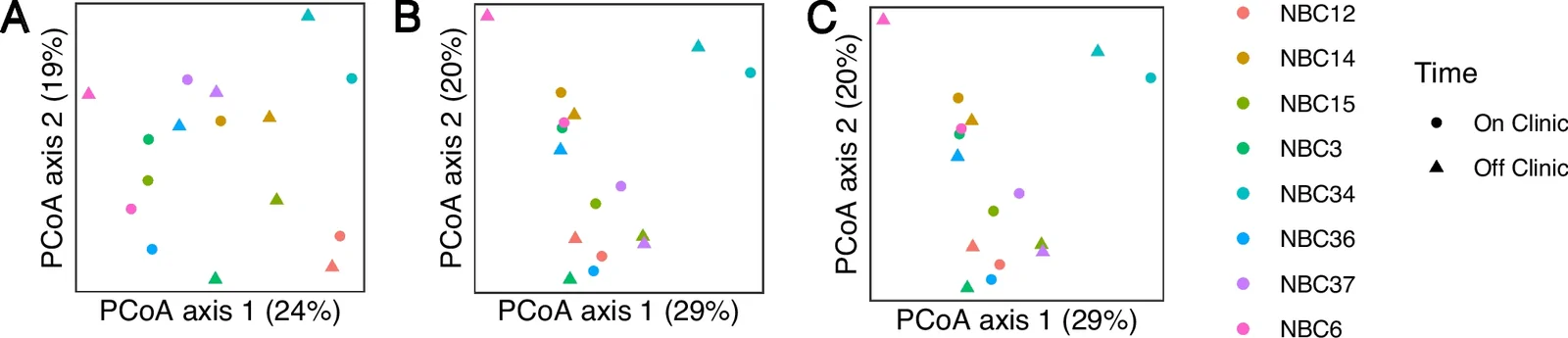
Principal coordinates analysis plots depicting Bray–Curtis beta diversity metrics of the fecal (**A**) and nasal (**B**) microbiota and fecal resistome (**C**) of veterinary personnel from a large animal academic hospital when they were on vs away from (off) clinics

**Fig. 8:**
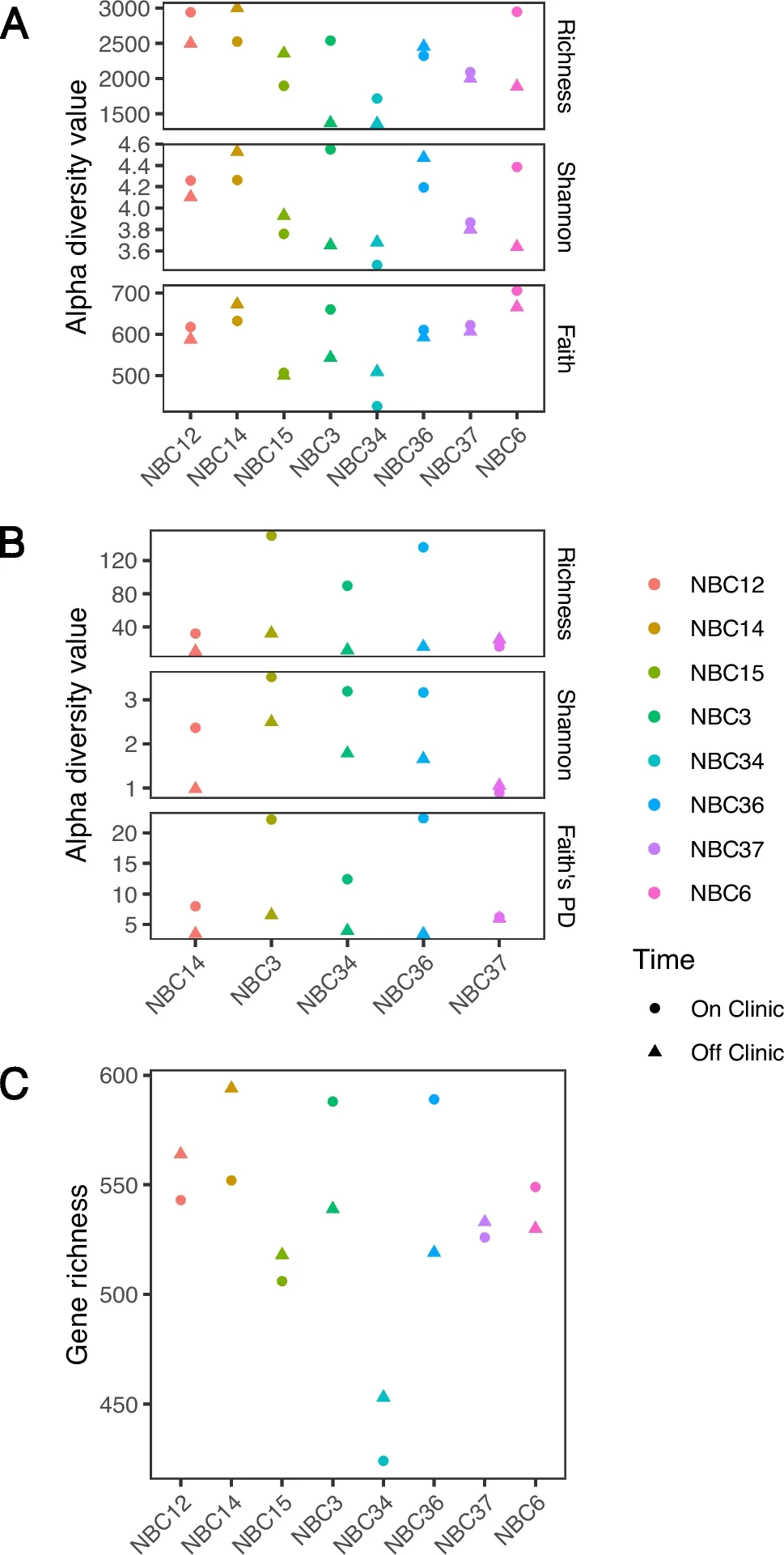
Alpha diversity of the fecal microbiome (**A**), nasal microbiome (**B**) and fecal resistome (**C**) of veterinary personnel from a large animal academic hospital when they were on vs away from (off) clinics

No significant difference in the overall composition (beta diversity) of the fecal (Fig. 7A) and nasal microbiota (Fig. 7B) or of the fecal resistome (Fig. 7C) were noted between on and off clinics samples. There was variable distance between on/off samples across the 8 participants, with some experiencing minimal change and others larger differences. Only one taxon in the nasal microbiota – *Actinobacteriota Cutibacterium*—was found at significantly lower levels when participants were off clinics (p = 0.013). No significant differences in alpha diversity of the fecal microbiome (Fig. 8A) or resistome (Fig. 8C) were noted between on- and off-clinic samples. However, the alpha diversity of the nasal microbiome (Fig. 8B) decreased significantly (p = 0.04 for Shannon diversity, p = 0.015 for Faith’s phylogenetic diversity) between on- and off-clinic samples.

## Discussion

The goal of this study was to understand the extent to which veterinary personnel carry AMR organisms and to assess the contribution of work site and occupational clinical exposures to the presence of AMR – both in terms of carriage of AMR organisms and the composition of their resistome. We found that carriage rates of *S. aureus* and ESCR-*E. coli* in veterinary personnel were similar to those documented in the general population (Graham et al. 2006; Kim et al. 2019; Woerther et al. 2013; Karanika et al. 2016; Bezabih et al. 2022) and in healthcare workers (Dulon et al. 2014; Peters et al. 2019; Badinski et al. 2024). However, rates of MRSA were substantially higher in veterinary personnel (18.5% at the academic veterinary hospital, 12.5% among private practice personnel) compared to 1–2% in the general public (Graham et al. 2006) and 5% in healthcare workers (Dulon et al. 2014)). The higher prevalence of MRSA carriage among NBC participants could be associated with contact with large/farm animals, a known risk factor for nasal MRSA carriage (Chen and Wu 2021), but this is only one possible explanation. Consistent with the hypothesis of clinical exposures influencing AMR carriage risk, we observed that administering antibiotics to patients and owning pets receiving antibiotics were strong predictors of carriage with MRSA.

While studies have shown that exposure to farm animals can alter the microbiome and resistome relative to no exposure (Tong et al. 2022; Wang et al. 2023; Xiong et al. 2023), the extent to which specific veterinary hospitals shape the microbiomes/resistomes of personnel working in these sites is unknown. We hypothesized that workers exposed to the same worksite would have more similar microbiota than workers from different work sites. Overall, we found modest support for this hypothesis. While substantial differences between the core composition of the microbiota in workers from different hospitals were not observed, several findings support a subtle but consistent effect of the hospital site on the microbiome/resistome. First, microbial composition differed significantly by work location when using unweighted UniFrac beta diversity (PERMANOVA p < 0.05, R^2^ = 0.02), but not with weighted UniFrac, Bray–Curtis, or Jaccard metrics. Unweighted UniFrac emphasizes the presence or absence of phylogenetically distinct taxa**,** while the other metrics are driven more by shared taxa and their relative abundances. This suggests that the work environment may influence the presence or absence of low-abundance or phylogenetically distinct taxa, rather than altering the overall dominance structure the microbiota. Second, we found that fecal samples from NBC participants and from the NBC environment shared a subset of microbial taxa, including some common gut commensals, that are not seen to the same degree as in samples from PP participants. Third, while we found that the number of distinct taxa (richness) and dominance structure (Shannon) of participants’ microbiota were not significantly different across groups, we did find that Faith’s PD, which reflects the phylogenetic diversity of the taxa that are present, differed by work site. While the effect size was small (2% of variance explained according to PERMANOVA), these findings nevertheless suggest a subtle ecological influence of workplace exposures on the microbiome. Because no differences in alpha diversity were noted and because the taxa that were found to be significantly different across work sites were commensals and butyrate producers (e.g., *Faecalibacterium prausnitzii* and *Anaerostipes hadrus),* the effect of work site is likely to be strictly ecological, and not associated with dysbiosis or increased carriage of pathogenic organisms. Moreover, it is likely that non-work-related factors in addition to normal inter-person variability in the composition of the microbiome, which can account for more than 50% of gut microbiome variation (Procházková et al. 2024), are stronger influencers of the microbiome composition and diversity. For example, we found that having dogs at home and a greater numbers of pets was significantly associated with decreased microbiome diversity; it is unclear why this is the case, as other studies have found pet ownership to be associated with more diverse microbiota (Song et al. 2013; Tun et al. 2017). This further supports the likelihood that workplace effects, if present, are layered onto a broader background of host and household influences.

While we did not find that direct clinical exposure to patients (vs. no patient contact) substantially impacted the composition of the microbiome/resistome, we did find that exposure to patients with diarrhea and/or reflux was associated with decreased microbiome diversity, suggesting that certain higher-risk clinical encounters may have greater effects than patient contact in general. Additionally, four AROs were found at significantly higher levels in participants with clinical/patient exposures than in participants with none. Notably, these AROs (*tet(T), dfrF,* and *vanS/vanH*) were class-specific resistance genes or operon components, which are more likely to be associated with specific resistant taxa or horizontally acquired resistance loci rather than intrinsic multidrug efflux or permeability mechanisms that are carried by a wide variety of bacteria. The enrichment of these AROs could be consistent with clinical exposure, where routine use of antibiotics occurs: indeed, *tet(T)* and *dfrF* are associated with resistance to tetracyclines and trimethoprim, respectively, which are the two most commonly orally administered antibiotics at the large animal academic hospital (Redding et al. 2019). However, these associations were limited in number and should be interpreted with caution given the exploratory nature of the analysis. Notably, vancomycin is never used at any of the hospitals, so the reasons for increases in the *vanS/vanH* genes is unlikely to be related to clinical exposures.

In contrast, ARG families that differed by site and clinical exposures were mostly multidrug efflux and porin families. These families are widely distributed across many bacterial taxa and are often chromosomally encoded (Li 2016), making them more likely to track differences in the underlying microbial community rather than targeted acquisition of specific resistance genes (Nesme et al. 2014). The lack of difference in beta diversity of the resistome across sites further suggests that most participants shared a broadly similar resistome backbone, even though a few individual AROs or broad families differed at low levels.

Given our hypothesis that occupational clinical exposures would be associated with higher likelihood of AMR carriage and resistome composition, we hypothesized that time away from clinics would result in decreased likelihood of carriage and alterations or reductions in the resistome. With the caveat that the number of participants who submitted off-clinic samples was very small, we did not note any findings consistent with our initial hypothesis. No participants carried ESCR-*E. coli* at any time points, while three patients who did not originally carry *S. aureus* did so after time away. This could simply reflect the transient and intermittent nature of *S. aureus* carriage, variability in the sensitivity of cultures, or it could reflect exposures encountered at home that are associated with *S. aureus* carriage, such as contact with owned horses. Similarly, we did not observe a reproducible directional shift in microbiome or resistome diversity after time away from clinics. Only one nasal taxon, *Cutibacterium*, a common skin and nasal commensal, was found at lower levels off clinic, suggesting at most a subtle shift in colonization by a common commensal rather than a change involving a major pathogenic organism. It is also possible that the time away from the clinics (1–2 weeks) was not sufficient to induce any consistent changes in organism carriage or microbiome composition. For example, patients can remain colonized with MRSA and certain Enterobacterales for weeks-to-months following hospital discharge (Shenoy et al. 2014). It is unknown if the same is true in non-hospitalized healthy workers. More data are required to elucidate the effect – if there is one – of time away from the clinical environment on AMR carriage and the resistome.

Overall, our findings suggest that the hospital environment may exert a modest influence on the microbiome and resistome of veterinary personnel, but that this effect is subtle and not consistently detectable across analytic approaches. A stronger signal was seen in the elevated prevalence of MRSA carriage among veterinary personnel. One possible explanation for this contrast is that targeted culture can capture specific clinically important carriage states, whereas the broader microbiome and resistome are dominated by background commensal structure and therefore may show only weak occupational signatures in cross-sectional sampling. Another possible explanation is that veterinary occupational risk is not a single exposure, but more likely a gradient consisting of exposures to different species of animals, environments/clinical services with gradients of hygiene practices, and procedures that are heterogeneous across participants.

This study has several limitations. While we did include a group of hospital workers without patient contact in our sampling population, the inclusion of a true control group with no exposure to the veterinary environment could have strengthened the implications of our findings related to an occupational impact on the microbiome. The study’s cross-sectional design also limits inference about the persistence of carriage and resistome differences, and single time-point sampling might miss transient or intermittent carriage. In contrast, the microbiome and resistome tend to be relatively stable (Mehta et al. 2018; Chen et al. 2021); thus, even a single sampling can provide valuable information on their composition, especially relative to that of other participants. However, we acknowledge that slight changes in sample handling could have affected the composition of the microbiome and resistome. Because of small and unbalanced subgroups, it was not always possible to identify factors that impact the microbiome and resistome and carriage risk. Nevertheless, these findings provide preliminary evidence that veterinary personnel may experience elevated carriage of certain AMR organisms, particularly MRSA, and that workplace environment may have a subtle effect on the microbiome and resistome. Larger longitudinal studies will be needed to determine whether these patterns reflect persistent occupational acquisition, transient exposure, or broader differences in host and environmental ecology.

## Supplementary Information

Below is the link to the electronic supplementary material.

Supplementary file1 (TSV 25 KB)

Supplementary file2 (XLSX 9 KB)

Supplementary file3 (XLSX 10 KB)

## Data Availability

Shotgun metagenomic data are available as BioProject ID: PRJNA1474380. Metadata provided as supplementary file 1.

## Declarations

Declaration of generative AI and AI-assisted technologies in the manuscript preparation process: During the preparation of this work, the authors used ChatGPT (OpenAI) to assist with developing the manuscript outline and generating preliminary draft text. All AI-generated content was critically reviewed, substantially revised, and edited by the authors, who take full responsibility for the final content of the manuscript.
